# Associations of social determinants of health on likelihood of systemic hormone therapy use in midlife women

**DOI:** 10.1186/s13293-025-00720-9

**Published:** 2025-06-01

**Authors:** Juliana M. Kling, Anna E. Abraham, Ekta Kapoor, Kristin Cole, Mariam Saadedine, Chrisandra Shufelt, Stacey J. Winham, Stephanie S. Faubion

**Affiliations:** 1 Mayo Clinic Center for Women’s Health, Mayo Clinic, 200 First St. SW, Rochester, Rochester, Minnesota, MN 55905 USA; 2 Division of Women’s Health Internal Medicine, Mayo Clinic, 13737 N. 92nd St. Scottsdale, Scottsdale, Arizona, AZ 85260 USA; 3 University of Arizona College of Medicine, 475 N 5th St, Phoenix, Phoenix, Arizona, AZ 85004 USA; 4 Division of Endocrinology, Diabetes, Metabolism and Nutrition, Mayo Clinic, 200 First St. SW, Rochester, Rochester, Minnesota, MN 55905 USA; 5 Department of Quantitative Health Sciences, Mayo Clinic, 200 First St. SW, Rochester, Rochester, Minnesota, MN 55905 USA; 6 Division of General Internal Medicine, Mayo Clinic, 4500 San Pablo Road, Jacksonville, Jacksonville, Florida, FL 32224 USA

**Keywords:** Social determinants of health, Menopausal hormone therapy, Menopause care, Women’s health, Midlife women, Health equity

## Abstract

**Objective:**

Social determinants of health (SDOH) can have a significant impact on women’s health and quality of life. Little is known about the impact of SDOH during menopause, and whether certain SDOH impact the likelihood of using systemic hormone therapy (HT). Our objective was to evaluate the impact of SDOH on the likelihood of HT utilization among midlife women.

**Design:**

Midlife women between the ages of 45–60 years were surveyed about their menopause experience between March and June of 2021. The questionnaire included information on medications used to treat menopause symptoms. From the electronic medical record demographic information and self-reported SDOH data were obtained, including the amount of exercise/physical activity, whether the participants felt stressed, social interactions, abuse in the last year, ability to pay for basics, diet, alcohol intake, smoking status, and whether participants had regular dentist visits. SDOH were compared between using/not using HT currently.

**Results:**

One thousand nine hundred and eighty-eight women aged 45–60 years who received primary care at one of four geographic Mayo Clinic sites completed the survey and filled out SDOH questions within 2 years. Women were 54.4 years of age on average (SD 4.2), with a mean BMI of 30.2 (SD 7.5), and a majority White (97%). 258 (13.0%) women were currently using HT. In univariate analysis, women were less likely to be using HT if they had higher BMI (per 1 kg/m^2^ increase, OR = 0.97, 95% 0.95–0.99, *p* = 0.002) were unpartnered (OR = 0.66, 95% CI 0.45–0.99, *p* = 0.04) had lower education (compared to post graduate studies, high school graduate/GED or less: OR = 0.45, 95% CI 0.24–0.85, *p* = 0.01; some college/2 year degree: OR = 0.69, 95% CI 0.49–0.96,*p* = 0.03), or were a smoker (compared to those who never smoked, current smoker: OR = 0.38, 95% CI 0.18–0.83, *p* = 0.02; former smoker: OR = 0.71, 95% CI 0.52–0.96, *p* = 0.03). Women who used extra virgin olive oil as main fat in diet were more likely to be using HT (OR = 1.46, 95% CI 1.10–1.94, *p* = 0.009). No other SDOH were associated with HT.

**Conclusion:**

Certain SDOH were associated with HT use for menopause treatment. Favorable SDOH likely correlate with better access to menopause care. To assure equitable menopause treatment for all women, clinicians should evaluate and address SDOH with their midlife women patients.

## Abstract (plain language summary)

Social determinants of health (SDOH) are known to have significant impacts on women’s health. However, little is known about the impact of SDOH on menopause. This study evaluates the impact of SDOH on the likelihood of menopausal hormone therapy (HT) use in midlife women. Women 45–60 years old were surveyed about their menopause experience, including medications used to treat their menopause symptoms. Self-reported SDOH data were extracted from the electronic medical record, and SDOH were compared between those who were currently using HT and those who were not using HT. The results of this study demonstrate that several protective SDOH factors are associated with a higher likelihood of HT use. These findings are important because they indicate that in order to ensure equitable access to menopause care, clinicians should be evaluating for and addressing SDOH with their midlife women patients.

## Highlights

In this study of a predominately White population, several SDOH factors were associated with increased menopausal hormone therapy use.

Protective SDOH such as, being partnered, having higher education, and using extra virgin olive oil, correlated with higher likelihood of menopausal hormone therapy use.

To assure equitable menopause treatment for all women, clinicians should evaluate and address SDOH with their midlife women patients in a culturally responsive manner.

## Background

Menopause affects women worldwide with an estimated 1.3 million women entering menopause each year in the US [1] at a mean age of 52 years [2]. Symptoms are common during menopause with as many as 80% of women reporting vasomotor symptoms [3, 4]. Menopause symptoms include hot flashes, night sweats, fatigue, muscle aches, sleep disturbance, and vaginal dryness [5, 6, 7], which can negatively impact women’s quality of life, as well as lead to adverse work outcomes [8].

Menopausal hormone therapy (HT) is the most effective treatment available for vasomotor symptoms (VMS) [9]. Furthermore, HT is effective for the treatment of the genitourinary syndrome of menopause (GSM) and prevention of osteoporosis [9]. Although use of HT declined following the Women’s Health Initiative (WHI) trials in 2002, with prevalence of HT use in 2010 dropping to 4.7% [10], current guidelines support use of HT for treatment of menopause-related symptoms in women who are under the age of 60 years or are within 10 years of their final menstrual periods [9]. However, most women experiencing menopause-related symptoms are not using HT. In a survey of postmenopausal women who reported experiencing vasomotor symptoms (VMS) in the last 12 months, 81% of participants in US had never received HT [5]. Furthermore, 50% of women in the US experiencing menopausal symptoms delayed seeking care for over 6 months [7]. The low rates of HT use for menopause-related symptoms and the delay in seeking care to address symptoms are likely influenced by multiple factors, and one of those could be social determinants of health (SDOH).

According to the World Health Organization, SDOH are defined as “conditions in which people are born, grow, work, live, and age, and the wider set of forces and systems shaping the conditions of daily life” [11]. It is known that SDOH impact women’s health in several ways. For example, housing insecurity and food insecurity in women are linked with worse mental health outcomes [12, 13], higher risk of chronic diseases [12, 14], and worse pregnancy outcomes [12, 13]. Additionally, women with higher levels of neighborhood poverty had lower anti-mullerian hormone (AMH) levels, indicating decreased ovarian reserve [15]. While there is evidence that lifestyle, family support, employment, socioeconomic status (SES), and marital status can impact age of menopause onset and menopause-related symptoms [16], the overall impact of SDOH during menopause is not well understood. Furthermore, it is not known whether the likelihood of using HT in menopausal women is related to SDOH.

This study aims to evaluate the impact of various SDOH on the likelihood of using HT in midlife women. The objectives of this study were to identify SDOH factors present in midlife women receiving primary care at one of four geographic locations of the Mayo Clinic and to determine whether these SDOH factors were associated with likelihood of HT use.

## Patients and methods

### Study design and participants

Midlife women receiving primary care at Mayo Clinic (Rochester, MN; Scottsdale, AZ; Jacksonville, FL; and Mayo Clinic Health System, NW WI) between the ages of 45–60 years in this cross-sectional study were surveyed about their menopause experience between March and June of 2021. The questionnaire included information on medications used to treat menopause symptoms. The study was approved by the Mayo Clinic Institutional Review Board.

### Outcome measures

#### SDOH factors

Demographic information (age, body mass index, race/ethnicity, education) and self-reported SDOH data was obtained from the electronic medical record, including amount of exercise/physical activity, whether the participants felt stressed, social interactions (attendance to clubs or organizations, talk on the telephone with friends/relatives), abuse in the last year, ability to pay for basics, diet (daily fruits and vegetables), alcohol intake, smoking status, and whether or not participants had regular dentist visits.

#### Use of HT

As part of the study questionnaire, participants were asked to report their current use of systemic hormone therapy for treatment of menopause-related symptoms.

### Statistical analyses

#### Data analyses

Descriptive statistics are reported as mean and standard deviation for continuous data and as frequencies and percentages for categorical data. Univariate logistic regression analysis was used to measure the association between SDOH and HT use. Associations were summarized using odds ratios (OR) and 95% confidence intervals (CI). Firth’s correction was used where data was sparse.

## Results

### Participants

A total of 32,469 surveys were sent and 5219 responses were received (16.1%); 1988 (38.1%) of participants who completed the survey also had completed SDOH information in the electronic medical record within two years of taking the survey and were included in the study.

Demographic characteristics of the survey participants in this study are summarized in Table 1. Women were 54.4 years of age on average (SD 4.2), with a mean BMI of 30.2 kg/m^2^ (SD 7.5), and a majority being White (97.1%) and married (83.5%). Of the total women in included in the study, 258 (13.0%) reported current systemic hormone use for menopause.

**Table 1:** Participant demographic and social determinant of health information

Systemic Hormone Therapy for Menopause						
	Total (*N* = 1988)	Yes^a^ (*N* = 258)	No^a^ (*N* = 1730)	Odds Ratio (95% CI)	χ^2^	p value
**Age (years)**, Mean (SD)	54.4 (4.2)	54.2 (4.0)	54.5 (4.2)	0.98 (0.95–1.01)	1.25	0.26
**BMI (kg/m**^**2**^**)**^**b**^, Mean (SD)	30.2 (7.5)	28.8 (6.5)	30.4 (7.7)	0.97 (0.95–0.99)	10.01	**0.002**
**Partner status**						
Married/Partnered	1659 (84%)	226 (14%)	1433 (86%)	Reference		
Single/widowed/separated/divorced	327 (17%)	31 (9%)	296 (91%)	0.66 (0.45–0.99)	4.11	**0.043**
**Race**						0.11
White	1915 (97%)	254 (13%)	1661 (87%)	Reference		
American Indian/Alaskan Native	5 (0.3%)	0 (0%)	5 (100%)	0.59 (0.03–14.19)	0.10	0.75
Asian	20 (1%)	0 (0%)	20 (100%)	0.16 (0.01–2.83)	1.57	0.21
Black or African American	17 (0.9%)	0 (0%)	17 (100%)	0.19 (0.01–3.38)	1.29	0.26
Native Hawaii/Pacific Islander	1 (0.1%)	0 (0%)	1 (100%)	2.49 (0.03–200.00)	0.17	0.68
Other	15 (1%)	4 (27%)	11 (73%)	2.56 (0.82–7.93)	2.63	0.10
**Education**						**0.032**
High school graduate/GED or less	150 (8%)	12 (8%)	138 (92%)	0.45 (0.24–0.85)	5.99	**0.014**
Some College or 2 year degree	651 (33%)	76 (12%)	575 (88%)	0.69 (0.49–0.96)	4.80	**0.028**
4-year college graduate	655 (33%)	85 (13%)	570 (87%)	0.78 (0.56–1.08)	2.31	0.13
Post graduate studies	528 (27%)	85 (16%)	443 (84%)	Reference		
**Do you feel stress these days?**						0.87
Not at all	286 (14.6%)	40 (14.0%)	246 (86.0%)	Reference		
Only a little	693 (35.3%)	83 (12.0%)	610 (88.0%)	0.84 (0.56–1.26)	0.74	0.39
To some extent	630 (32.1%)	85 (13.5%)	545 (86.5%)	0.96 (0.64–1.44)	0.04	0.84
Rather much	185 (9.4%)	22 (11.9%)	163 (88.1%)	0.83 (0.48–1.45)	0.43	0.51
Very much	171 (8.7%)	23 (13.5%)	148 (86.5%)	0.96 (0.55–1.66)	0.03	0.87
**Within the past 12 months, you worried that your food would run out before you got money to buy more**						0.11
Never true	1882 (96%)	250 (13%)	1632 (87%)	Reference		
Sometimes true	64 (3%)	5 (8%)	59 (92%)	0.60 (0.25–1.47)	1.24	0.26
Often true	19 (1%)	0 (0%)	19 (100%)	0.17 (0.01–2.99)	1.48	0.22
**Smoking Status**						**0.006**
Current Smoker	115 (6%)	7 (6%)	108 (94%)	0.38 (0.18–0.83)	5.89	**0.015**
Former Smoker	558 (28%)	60 (11%)	498 (89%)	0.71 (0.52–0.96)	4.82	**0.028**
Never Smoked	1313 (66%)	191 (15%)	1122 (85%)	Reference		
**How often do you have a drink containing alcohol?**						0.61
Never	334 (16.9%)	41 (12.3%)	293 (87.7%)	Reference		
Monthly or less	596 (30.2%)	67 (11.2%)	529 (88.8%)	0.91 (0.60–1.37)	0.22	0.64
2–4 times a month	502 (25.5%)	61 (12.2%)	441 (87.8%)	0.99 (0.65–1.51)	0.003	0.96
2–3 times a week	384 (19.5%)	62 (16.1%)	322 (83.9%)	1.38 (0.90–2.11)	2.17	0.14
4 or more times a week	156 (7.9%)	23 (14.7%)	133 (85.3%)	1.24 (0.71–2.14)	0.57	0.45
**Physical activity**						0.29
Inactive (0 min/week)	278 (14.2%)	29 (10.4%)	249 (89.6%)	Reference		
Insufficiently Active (10–140 min/week)	1661 (84.7%)	226 (13.6%)	1435 (86.4%)	1.35 (0.90–2.04)	2.09	0.15
Sufficiently Active (150 + min/week)	23 (1.2%)	2 (8.7%)	21 (91.3%)	0.82 (0.18–3.67)	0.07	0.79
**On average, how many servings of fruit and/or vegetables do you eat a day?**						**0.038**
0–1	342 (17%)	36 (11%)	306 (89%)	Reference		
2–3	1144 (58%)	148 (13%)	996 (87%)	1.25 (0.85–1.84)	1.31	0.25
4–5	400 (20%)	60 (15%)	340 (85%)	1.49 (0.96–2.32)	3.19	0.074
6–7	68 (3%)	13 (19%)	55 (81%)	2.04 (1.02–4.08)	4.11	**0.043**
8 or more	26 (1%)	0 (0%)	26 (100%)	0.16 (0.01–2.80)	1.58	0.21
**Do you use extra virgin oil as your main source of fat in your diet?**						
N	738 (37%)	77 (10%)	661 (90%)	Reference		
Y	1245 (63%)	181 (15%)	1064 (85%)	1.46 (1.10–1.94)	6.83	**0.009**
**How hard is it for you to pay for the very basics like food, housing, medical care, and heating?**						0.19
Not hard at all	1428 (73%)	182 (13%)	1246 (87%)	Reference		
Not very hard	379 (20%)	57 (15%)	322 (85%)	1.22 (0.88–1.68)	1.45	0.23
Somewhat hard	113 (6%)	10 (9%)	103 (91%)	0.69 (0.36–1.34)	1.20	0.27
Hard	20 (1%)	3 (15%)	17 (85%)	1.37 (0.42–4.47)	0.27	0.61
Very hard	19 (1%)	0 (0%)	19 (100%)	0.18 (0.01–3.13)	1.40	0.24
**Within the past 12 months, the food you bought just didn’t last and you didn’t have money to get more**						**0.045**
Never true	1901 (97%)	251 (13%)	1650 (87%)	Reference		
Sometimes true	47 (2%)	1 (2%)	46 (98%)	0.21 (0.04–1.10)	0.38	0.065
Often true	8 (0.4%)	0 (0%)	8 (100%)	0.39 (0.02–7.98)	3.41	0.54
**Social connections** ^**c**^						0.43
Socially Isolated	213 (12.0%)	22 (10.3%)	191 (89.7%)	0.82 (0.49–1.34)	0.64	0.42
Moderately Isolated	438 (24.7%)	64 (14.6%)	374 (85.4%)	1.21 (0.85–1.73)	1.12	0.29
Moderately Integrated	484 (27.3%)	66 (13.6%)	418 (86.4%)	1.12 (0.79–1.59)	0.38	0.54
Socially Integrated	638 (36.0%)	79 (12.4%)	559 (87.6%)	Reference		
**Intimate partner violence** ^**d**^						
At Risk	68 (3.6%)	11 (16.2%)	57 (83.8%)	1.32 (0.68–2.55)	0.67	0.41
Not at Risk	1831 (96.4%)	234 (12.8%)	1597 (87.2%)	Reference		

Bolded numbers are statistically significant

^a^ Percentages are reported as row percentages

^b^ Missing in 39 (5 with systemic hormone therapy and 34 without)

^c^ Patients receive 1 point for each of the following 4 components:

1) Being married or living with a partner at the time of assessment,

2) Having 3 or more interactions per week with other people. This is the sum of the interactions specified in the following 2 questions: (a) “In a typical week, how many times do you talk on the telephone with family, friends, or neighbors?” and (b) “How often do you get together with friends or relatives?“,

3) Attending church or religious services at least once per year,

4) Reporting membership or participation at least once per year in a club or organization such as a church group, union, fraternal or athletic group, or school group

0 or 1 points - Socially Isolated, 2 points - Moderately Isolated, 3 points - Moderately Integrated, 4 points - Socially Integrated

^d^ “At Risk” if they answered “Yes” to any of the following:

a) Within the last year, have you been humiliated or emotionally abused in other ways by your partner or ex-partner?

b) Within the last year, have you been afraid of your partner or ex-partner?

c) Within the last year, have you been raped or forced to have any kind of sexual activity by your partner or ex-partner?

d) Within the last year, have you been kicked, hit, slapped, or otherwise physically hurt by your partner or ex-partner?

### HT and SDOH

Overall, in univariate analysis, women were less likely to be using HT if they had higher BMI (per 1 kg/m^2^ increase, OR = 0.97, 95% 0.95–0.99, *p* = 0.002) were unpartnered (OR = 0.66, 95% CI 0.45–0.99, *p* = 0.04) education (compared to post graduate studies, high school graduate/GED or less: OR = 0.45, 95% CI 0.24–0.85, *p* = 0.01; some college/2 year degree: OR = 0.69, 95% CI 0.49–0.96,*p* = 0.03), or were a smoker (compared to those who never smoked, current smoker: OR = 0.38, 95% CI 0.18–0.83, *p* = 0.02; former smoker: OR = 0.71, 95% CI 0.52–0.96, *p* = 0.03). Women who used extra virgin olive oil as main fat in diet were more likely to be using HT (OR = 1.46, 95% CI 1.10–1.94, *p* = 0.009). Table 1 indicates all results including statistically significant findings. No other SDOH were statistically significantly associated with HT.

## Discussion

In this study of predominantly White women with health insurance, favorable SDOH were associated with higher likelihood for HT use. This is in keeping with prior literature that has shown a relationship between SDOH and a women’s health overall. For example, women who experience both housing and food insecurity are more likely to experience poor mental health outcomes, and are at higher risk of chronic disease, and poorer pregnancy outcomes than women who do not experience those insecurities [12, 13, 14]. In the current study, protective SDOH correlated with higher likelihood of HT use. It is possible that these factors themselves made it more likely she is on HT, or instead it is because of the protective SDOH she has better access to care.

While the relationship between SDOH and menopause is not fully understood, there are prior studies that align with our findings demonstrating the impact of SDOH on experiences in menopause. Documented risk factors for higher reported rates of VMS during menopause include lower education, higher BMI, smoking, and symptoms of anxiety [17]. Smoking may be considered a relative contraindication to HT prescribing as well and thus impact HT prescribing likelihood. VMS duration has also been found to be longer in women with lower educational levels, greater perceived stress, and more depressive and anxiety symptoms [3]. Overall, lifestyle, family support, employment, SES, and marital status has been shown to impact menopause-related symptoms [16]. Furthermore, a study examining HT prescribing patterns in the UK found that there was an 18% lower rate of HT prescribing in primary care practices with low socioeconomic status (SES) patient populations compared to those with patients of higher SES [18]. Similarly, we found that several SDOH factors, such as partner status, education level, and food insecurity impacted women’s likelihood of using HT. In other words, women with more unfavorable SDOH, referred to as SDOH exposure, are expected to have a higher burden of menopause symptoms, and are less likely to have access to HT. This may partially explain the increased duration of menopausal symptoms in women with higher SDOH exposure.

SDOH exposure is highest among non-White populations in the United States. According to a 2022 survey, receiving food stamps or Supplemental Nutritional Assistance Plan (SNAP) was most prevalent among Black and American Indian or Alaska Native adults [19]. Food insecurity and housing insecurity were most prevalent among Native Hawaiian or other Pacific Islander adults, and uninsured status was most prevalent among Hispanic adults [19]. This study population predominantly consisted of White women (97.1%) with access to reliable primary care and health insurance, and the relationships identified were positive between favorable SDOH and likelihood of HT use. This suggests that for women of color and marginalized groups who are more likely to be exposed to unfavorable SDOH, the likelihood of access to menopause care and HT is lower. Unfortunately, these women are often excluded from retrospective studies and underrepresented due to limited access to care. Therefore, it is crucial to conduct more population-based studies to ensure better representation. It is plausible that some of the other SDOH outcomes evaluated in our study, such as stress, abuse and ability to pay for basic needs, may associate with lower HT access in marginalized populations.

Food deserts tend to have residents with lower incomes, lower educational levels, and higher poverty rates than areas not considered to be food deserts [20]. In our study, women with HT use were more likely to use extra virgin olive oil as their main fat. This can be interpreted as a marker of access to healthy foods as well as health literacy [21], as extra virgin olive oil has been demonstrated to have health benefits over other fats. However, the relatively higher cost compared to other fat options could be a barrier to individuals of lower SES living in food deserts. Additionally, women with HT use in our study had higher education level than those without HT use, which is directly associated with higher health literacy [22]. Given the history of negative messaging surrounding the safety of HT following publication of the initial Women’s Health Initiative trial results in 2002 [23], public opinion remains negative toward HT use for menopause symptoms. Health literacy is likely an important contributing factor for women who have access to HT and ultimately decide to use it as part of their treatment [24].

The influence of SDOH factors on HT access even in the current study’s predominantly White population demonstrates the importance of cultural agility in menopause care. In order to adequately address the differing experiences of menopause across diverse populations and to decrease disparities, SDOH should be considered in menopause healthcare. Culturally responsive care consists of cultural curiosity, respectful query, and connected care [25]. By using this framework clinicians can facilitate productive conversations about the various identities of their patients that could potentially impact their menopause experiences, including access to HT [25]. Additionally, recognizing the importance and diversity of the factors that influence menopause, including cultural identities, beliefs and attitudes, and socioeconomic factors, is an important step toward creating equity in menopause care [26]. A patient-centered approach that empowers individuals to take part in their own care and treatment can create a space for clinicians and patients to work together in addressing the SDOH factors that ultimately impact women’s experiences during menopause [26]. These are important considerations for reducing barriers to menopause care, including HT access.

The potential limitations of this study include the cross-sectional study design and reliance on self-reported SDOH factors. Given the 16.1% response rate, sampling bias could have impacted the results as it is possible that women who had more extreme experiences of menopause were more likely to respond. The way the data was gathered did not allow for comparison between responders and non-responders. The study population consisted of a low percentage of racially and ethnically diverse individuals. Furthermore, this cohort was made up entirely of women receiving care at a large medical institution. These factors could have underestimated the impact of SDOH on HT access given that these women were less likely to experience SDOH [19] and already had access to care, including specialty menopause care. Future research is needed to explore the impact of SDOH on usage of HT in more diverse populations, including those without regular access to healthcare. Additionally, it will be beneficial to explore contraindications to HT in women with favorable SDOH vs. women with exposure to SDOH to evaluate if women with unfavorable SDOH may have more risk factors or contraindications which ultimately exclude them from HT compounding limitations to care access.

## Conclusion

In this large study of predominantly White women with access to care, various SDOH were associated with current HT use for menopause treatment. It’s likely that favorable SDOH correlate with better healthcare access and access to menopause treatments. To assure equitable menopause treatment for all women, clinicians should evaluate and address SDOH with their midlife women patients in a culturally responsive manner. Follow up studies in diverse populations can provide further insight into these relationships.

## Data Availability

No datasets were generated or analysed during the current study.
